# HPV Detection in Breast Tumors and Associated Risk Factors in Northeastern Brazil

**DOI:** 10.3390/cells13131132

**Published:** 2024-06-29

**Authors:** Kamylla Conceição Gomes Nascimento, Bianca de França São Marcos, Pedro Henrique Bezerra Fontes, Beatriz Eda de Oliveira Isídio, Stephanie Loureiro Leão, Gabriel Romulo Parente da Silva, David Beltrán Lussón, Daffany Luana dos Santos, Lígia Rosa Sales Leal, Benigno Cristofer Flores Espinoza, Larissa Silva de Macêdo, Pedro Luiz de França Neto, Anna Jéssica Duarte Silva, Jacinto Costa Silva Neto, Vanessa Emanuelle Pereira Santos, Antonio Carlos de Freitas

**Affiliations:** Laboratory of Molecular Studies and Experimental Therapy, Department of Genetics, Federal University of Pernambuco, Av. Prof. Moraes Rego, 1235. Cidade Universitária Recife, Pernambuco, Recife 50670901, PE, Brazil; kamylla.conceicao@ufpe.br (K.C.G.N.); bianca.saomarcos@ufpe.br (B.d.F.S.M.); pedro.bezerrafontes@ufpe.br (P.H.B.F.); beatriz.eda@ufpe.br (B.E.d.O.I.); stephanie.lleao@ufpe.br (S.L.L.); gabriel.romulo@ufpe.br (G.R.P.d.S.); david.beltran@ufpe.br (D.B.L.); daffany.luana@ufpe.br (D.L.d.S.); ligia.leal@ufpe.br (L.R.S.L.); benigno.cristofer@ufpe.br (B.C.F.E.); larissa.smacedo@ufpe.br (L.S.d.M.); pedro.francaneto@ufpe.br (P.L.d.F.N.); anna.jessica@ufpe.br (A.J.D.S.); jacinto.costa@ufpe.br (J.C.S.N.); vanessa.emanuelle@ufpe.br (V.E.P.S.)

**Keywords:** breast cancer, sociodemographic, lifestyle, HPV16, E5 oncogene, triple-negative, invasive ductal carcinoma

## Abstract

Breast cancer risk factors include lifestyle, genetic–hormonal influences, and viral infections. Human papillomavirus (HPV), known primarily as the etiological agent of cervical cancer, also appears active in breast carcinogenesis, as evidenced in our study of 56 patients from northeastern Brazil. We assessed the clinical and sociodemographic characteristics, correlating them with various breast cancer tumor types. HPV detection involved amplifying the L1 region, with viral load measured using the E2/E6 ratio and viral activity indicated by E5 oncogene expression. Predominantly, patients over 56 years of age with healthy lifestyles showed a high incidence of invasive ductal carcinoma and triple-negative breast cancer. HPV was detected in 35.7% of cases, mostly HPV16, which is associated with high viral loads (80 copies per cell) and significant E5 expression. These results hint at a possible link between HPV and breast carcinogenesis, necessitating further studies to explore this association and the underlying viral mechanisms.

## 1. Introduction

Breast cancer is one of the most commonly diagnosed cancers worldwide and the leading cause of cancer-related mortality in women globally [1]. In 2022, according to the Global Cancer Observatory, approximately 2.3 million new cases of breast cancer were estimated worldwide, with approximately 670,000 associated deaths [2]. Considering statistical projections, there is an expected increase in breast cancer cases, with an estimated 3.19 million new cases projected by 2040 [3].

Breast cancer classification considers histological and molecular aspects of the disease. Histological classification categorizes tumors into invasive ductal carcinoma (IDC), responsible for about 50% to 80% of diagnosed cases; invasive lobular carcinoma (ILC); and ductal carcinoma in situ (DCIS) [4,5]. Molecular types of breast cancer include four main subtypes: luminal A, luminal B, HER2-enriched, and triple-negative. Luminal tumors represent 60–70% of cases and express estrogen and progesterone hormone receptors, HER2+ tumors overexpress the HER2 gene, and triple-negative tumors lack hormone receptors and HER2 expression and have high recurrence rates [6].

The prolonged use of contraceptive pills and hormone replacement therapy with estrogen may increase the risk of developing the disease [7]. However, other non-hormonal factors are also associated with the development of the disease, such as sedentary lifestyle, a high-fat diet, obesity, alcohol consumption, smoking, family history (BRCA1/2 gene mutations), and exposure to pollutants [8]. In recent years, studies have reported the Human Papillomavirus (HPV) as a possible risk factor; however, although HPV DNA has been detected in breast cancer samples, its relationship with mammary carcinogenesis remains controversial and unconfirmed [9].

The association between breast cancer and Human Papillomavirus (HPV) has been the subject of investigation in recent years [9]. HPV is the most common sexually transmitted virus in the population, capable of infecting epithelial cells in both mucosal and keratinizing skin [10,11]. Studies report the presence of the HPV genome in breast tumors; however, the frequency of virus detection in samples shows a wide variation, from 1.1 to 86% among different studies [12]. Some studies suggest that HPV infection may be related to the development of breast cancer, possibly due to the virus’s ability to integrate its DNA into the host cell genome, causing genetic instability [13].

Currently, more than 200 types of HPV are known, classified according to oncogenic risk. There are 20 types of high-risk HPVs (hr-HPV): 16, 18, 26, 31, 33, 35, 39, 45, 51, 53, 52, 56, 58, 59, 66, 67, 68, 70, and 82 [11,14,15], with type 16 being the most prevalent in the population of northeastern Brazil [16]. The oncogenic potential of HPV arises from the oncoproteins E5, E6, and E7, responsible for causing cellular transformations and interacting with pathways responsible for proliferation, angiogenesis, and cellular apoptosis [17].

The transmission of HPV to breast tissue is not fully understood; however, several mechanisms have been proposed. One hypothesis is viral dissemination from a preexisting genital infection, through the bloodstream or lymphatic system, to breast tissue [18]. Another hypothesis indicates the transmission of the virus between mothers and children during breastfeeding [19]. Furthermore, studies suggest that HPV transmission to the breast can occur from any oral site due to oral sexual practices, as well as through the nipple or microlesions in breast skin resulting from genital-to-breast sexual activity [12]. However, a comprehensive understanding of the mechanisms of HPV transmission to breast tissue still requires further research for elucidation.

HPV is the primary etiological agent of cervical cancer and is also implicated in the development of other cancers, including vulvar, vaginal, anal, and head and neck cancers. However, the virus’s activity in breast carcinogenesis remains inconclusive [20]. Therefore, this study aimed to identify the presence of HPV in breast tumor tissues of women from northeastern Brazil, as well as to characterize aspects related to other risk factors for breast carcinogenesis in breast cancer patients with and without virus infection.

## 2. Materials and Methods

### 2.1. Sample Population

This study included 56 individuals with a histological diagnosis of breast cancer, conducted in accordance with the World Health Organization (WHO) classification. Fresh breast tissue samples were collected between June 2012 and December 2015. Biopsies were obtained from regions with lesions indicative of neoplastic conditions and stored in RNA later solution. Women infected with HIV (Human Immunodeficiency Virus) or who were pregnant were excluded from the study.

This work was approved by the Research Ethics Committee of the Federal University of Pernambuco, Brazil, under the numbers CAAE: 28508614.9.0000.5208 and 28508614.9.0000.5208. The patients were informed about the research objectives and the procedures to be performed. All patients agreed to participate in this study, signed the Free and Informed Consent Form and completed a medical history. The medical history included sociodemographic and behavioral data.

### 2.2. DNA Extraction and HPV Detection

The biopsy samples were macerated in liquid nitrogen, and the DNA was extracted and purified using the DNeasy Blood & Tissue Kit (Qiagen GmbH, Hilden, Germany). All the samples were quantified using the NanoDrop™ Lite Spectrophotometer (Thermo Scientific, Waltham, MA, USA). The quality of the extracted DNA was confirmed by amplifying a fragment of the β-globin gene via the polymerase chain reaction (PCR), using the *primers* described in Table 1. HPV DNA detection was performed through PCR amplification using two sets of oligonucleotides. In the first step, we used the consensus and degenerate primer set MY09 and MY11 (Table 1), which bind to a conserved region of the HPV L1 gene, resulting in the amplification of a product approximately 450 bp in size. This was followed by Nested-PCR using the GP5 and GP6 oligonucleotides, generating a fragment of approximately 150 bp.

The PCR products were visualized on a 2% agarose gel stained with ethidium bromide. We used HPV-positive DNA samples from patients with cervical cancer, as well as a cloned HPV16 genome as positive controls for all the PCR reactions. Additionally, all tests were performed in triplicate by different handlers. 

### 2.3. Genotyping

Positive samples for HPV DNA were typed by sequencing from the GP5/6 oligonucleotides. The PCR products were sequenced using the fluorescent dideoxy-terminal method with the ABI PRISM BigDye™ Terminator Cycle Sequencing v 3.1 Ready Reaction kit (Applied Biosystems^®^, Foster City, CA, USA). The resulting sequences were analyzed using the programs Gap4 (version 4.0) and Pregap4 (version 1.5), which were used to construct the contigs of the obtained HPV DNA sequences. After the contigs were assembled, the BLAST program, available at http://blast.ncbi.nlm.nih.gov/Blast.cgi, was used for comparison with previously known HPV sequences.

### 2.4. Physical Status and Viral Load 

To determine the physical state and viral load of the most prevalent HPV type, real-time PCR (qPCR) reactions were performed using the Quantitect SYBR Green PCR kit (Sigma®, St. Louis, MO, EUA). The amplifications were conducted in a volume of 20 µL, containing 2× Power SYBR Green PCR Master Mix and oligonucleotides that amplify the E6 (*primer* forward—GAG AAA CTG CAA TGT TTC AGG ACC; *primer* reverse—TGTATAGTTGTTTGCAGCTCTGTGC) and E2 regions of HPV16 (*primer* forward—AAC GAA GTA TCC TCT CCT GAA ATT ATT AG; *primer* reverse—CCA AGG CGA CGG CTT TG; [24] at a concentration of 300 nM. The reactions were carried out using the Rotor Gene 6000 (Qiagen, Germantown, MD, USA) and Cycler 8800 (Agilent Scientific Instruments). 

To generate the standard curve, we used a series of dilutions (10^1 to 10^6) of the full-length HPV16 genome cloned into the pBR-322 vector. These dilutions served as standards for the calibration curve of the E2 and E6 genes. Additionally, we used DNA extracted from CaSki cell lines (400–6000 copies per cell) and SiHa cell lines (1–2 copies per cell) as positive controls for the reactions. These controls were used to verify the accuracy and reproducibility of the calibration curves estimated for the HPV load.

The samples were analyzed in duplicate after constructing the calibration curves for each gene. The viral load was determined based on the levels of the E6 gene, and the viral load values for each sample were expressed as the number of E6 copies in 50 ng of DNA [24]. Regarding the physical status of the virus, we considered HPV to be integrated into the host genome when the E2 gene was not detected. To distinguish between the episomal form and the mixed form (when both episomal and integrated forms are present), we calculated the E2/E6 ratio. The E2/E6 ratio values above 1 indicated a predominance of the episomal form [24].

### 2.5. RNA Extraction and cDNA Synthesis

RNA extraction was performed using liquid nitrogen and the Trizol protocol (Invitrogen®, Carlsbad, CA, USA), following the manufacturer's recommendations. For RNA purification, the samples were processed using the miRNeasy kit (Qiagen, Germantown, MD, USA). The integrity of the RNA was evaluated through RNA-specific electrophoresis, and the samples were quantified using the NanoDrop™ Lite Spectrophotometer (Thermo Scientific). Subsequently, 1 μg of RNA from each sample was subjected to reverse transcription using the miScript II RT kit (Qiagen, Germantown, MD, USA).

### 2.6. Quantitative Real-Time PCR (RT-qPCR) 

The RT-qPCR technique was employed to quantify the expression of the HPV E5 oncogene, Forwad: ACT GGC GTG CTT TTT GCT TTG and Reverse: GAC ACA GAC AAA AGC AGC GG [25]. The endogenous genes GAPDH and ACTB were used as internal standards for data normalization, allowing for the calculation of relative quantification values for all the evaluated targets. The Quantitect SYBR Green PCR kit (Sigma®) was used in the RT-qPCR reactions. The Rotor Gene 6000 (Qiagen, Germantown, MD, USA) was utilized as the thermal cycler, and each sample was analyzed in duplicate. The quantification cycle (Cq) values were converted to a ratio relative to the control and reference genes. The relative quantification data were generated following the qBase model [26], used for expression data analysis.

### 2.7. Statistical Analysis

We conducted statistical analyses to assess the association between the histopathological data and the presence of viral DNA in breast tissue. The chi-square test was used to compare the case and control groups. A *p*-value < 0.05 was considered statistically significant. The statistical analyses were performed using GraphPad Prism software version 9.0.0 (GraphPad Software, Inc., San Diego, CA, USA).

## 3. Results

### 3.1. Analysis of Clinical and Sociodemographic Characteristics

A frequence contingency analysis was performed on 56 breast cancer tumor samples, taking into account the clinical characteristics of the tumors as well as the sociodemographic characteristics of the patients (Table 2, Table 3 and Table 4). Breast cancer patients were evaluated based on their clinical and sociodemographic characteristics. More than 50% of patients were over 56 years of age, had a normal BMI (45.8%), were non-smokers, non-diabetic, and non-hypertensive. However, these data did not achieve statistical significance (Table 2).

In terms of patients’ gynecological status, the majority experienced menarche between 11–14 years (67.8%), menopause between 45–50 years (35.7%), their first pregnancy between 18–27 years (73.5%), with 1–3 children (50.0%). Additionally, over 55% of them breastfed and did not use hormonal contraception (53.6%) (Table 3).

When evaluating the breast cancer screening activity of the surveyed patients, a significant portion reported regular self-examination (78.6%) and mammograms (55.4%), with no family history of cancer (78.6%) (Table 4).

The histological and molecular characteristics of the tumors were mostly invasive ductal carcinoma (78.6%) and triple-negative (48.2%). Regarding HPV infection, the virus was detected in 35.7% of the tumors, all of which were HPV16, with the majority found in mixed form (65.0%) (Table 5)

### 3.2. Characteristics of HPV-Positive Samples

The HPV-positive samples were stratified by their histological type and the physical status of the virus (Table 6).

A substantial number of HPV-positive tumors were of the invasive ductal carcinoma type (75%), and a significant proportion of these tumor types exhibited HPV in its mixed form (66.6%). The second most detected tumor type, in which HPV was found, was ductal carcinoma in situ (20%), with half of them showing the virus in its integrated form and the other half in the mixed form. Only one of the HPV-positive tumors was of the invasive lobular carcinoma type, with the viral genome present in the mixed form.

The distribution of molecular breast cancer types was assessed among the HPV-positive samples across the different types of breast carcinoma. The triple-negative type was more frequent in invasive ductal carcinomas (77.7%). Only two HPV-positive tumors were of the ductal carcinoma in situ histological type, one was HER2-enriched, and the other was triple-negative. The single HPV-positive invasive lobular carcinoma was of the triple-negative molecular type (Table 7).

### 3.3. Evaluation of HPV Viral Load and E5 Protein Expression in Breast Tumors

**Figure 1:**
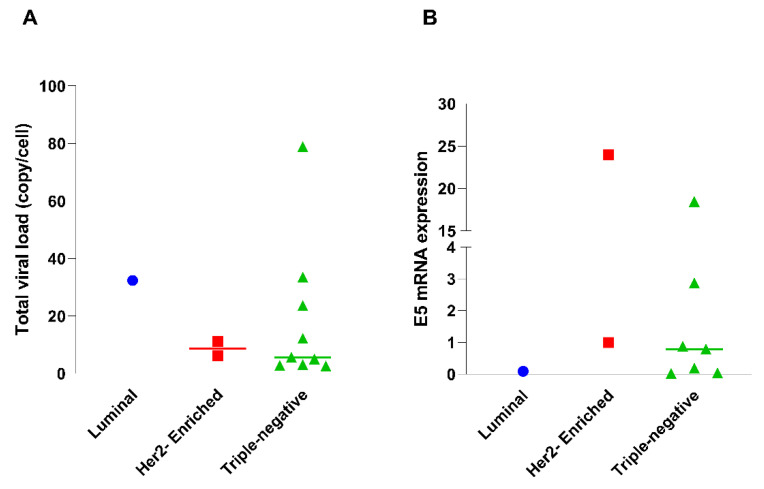
Distribution of viral load and E5 expression across tumor molecular subtypes. (**A**) Comparation of total viral load across different molecular subtypes of tumors: luminal, HER2-enriched, and triple-negative. (**B**). E5 mRNA expression across different molecular subtypes of tumors: luminal, HER2-enriched, and triple-negative.

The viral load and expression of the HPV16 oncoprotein E5 were evaluated concerning both the molecular types of breast cancer and the histological types of breast cancer (Figure 1A). The viral load of HPV16 found in the evaluated tumors was higher in the triple-negative molecular type, with one sample showing nearly 80 copies/cell (Figure 1A). However, regarding the expression of E5, this oncoprotein was more expressed in one of the HER2-enriched samples; however, HPV16 E5 also showed overexpression in triple-negative samples. Only in the luminal types did E5 show low expression (Figure 1B).

**Figure 2:**
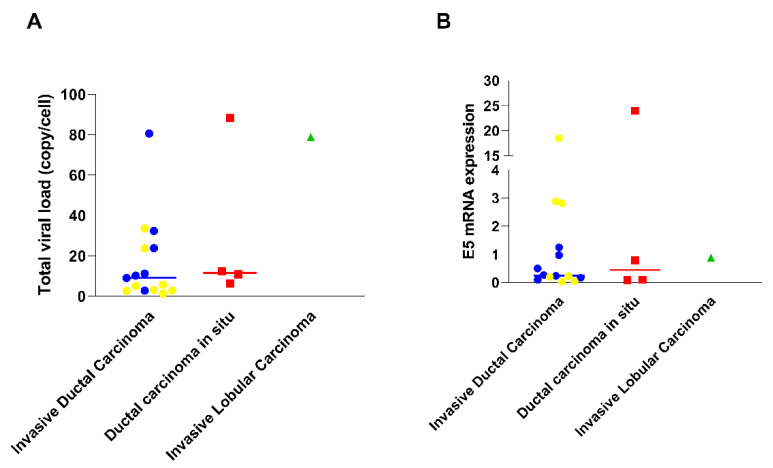
Distribution of viral load and E5 expression across tumor histological subtypes and molecular Subtypes. (**A**) Comparation of total viral load among different types of carcinomas: invasive ductal carcinoma, ductal carcinoma in situ, and invasive lobular carcinoma. (**B**) E5 mRNA expression among different types of carcinomas: invasive ductal carcinoma, ductal carcinoma in situ, and invasive lobular carcinoma. The samples highlighted in yellow represent triple-negative tumors.

The distribution of the samples concerning viral load across the different histological subtypes showed a higher number of virus copies per cell in ductal carcinoma in situ tumors (with over 80 copies/cell), followed by invasive ductal carcinoma, and invasive lobular carcinoma tumors (Figure 2A). However, when assessing E5 expression, it was observed that the oncoprotein was less expressed in invasive lobular carcinoma. Conversely, E5 was more expressed in one of the ductal carcinoma in situ samples followed by samples of the invasive ductal carcinoma type. When evaluating which molecular types were associated with histological types, we observed that triple-negative tumors (the most frequent molecular type in our samples) had a higher E5 expression when associated with invasive ductal carcinoma type (Figure 2B).

## 4. Discussion

Breast cancer is a global health issue associated with risk factors, including lifestyle, genetic, and demographic factors [27,28]. Studies report that age, family history, use of oral contraceptives, menopausal status, and genetic factors are significantly linked to breast cancer [29,30]. Here, we evaluated these characteristics in 56 breast cancer patients from northeastern Brazil. Approximately 80% of the population affected by breast cancer is over 50 years of age [31,32,33]. In our samples, over 50% of patients are over 56 years of age, as observed in other studies [29,34].

Prolonged exposure to estrogen, as occurs in women with early menarche and puberty, as well as late menopause, can significantly increase the risk of breast cancer due to prolonged hormone exposure [35]. Here, we observed that most breast cancer patients experienced menarche between 11–14 years and menopause between 45–50 years. A study comparing women with and without breast cancer found no influence of these characteristics on breast cancer development [29]. Conversely, the use of oral contraceptives was considered an influential factor in the development of this type of cancer. However, in our study, the majority of patients did not use hormonal contraception, suggesting that, in our patients, this characteristic did not have a significant influence.

Pregnancy and breastfeeding are factors considered protective against the development of breast cancer [36,37]. We observed that the majority of patients evaluated here have between one and three children, with breastfeeding present in more than 50% of them. This result reinforces the existence of a variety of risk factors involved in breast carcinogenesis, indicating that this characteristic did not have a protective effect in most samples evaluated here.

Family history is a commonly associated risk factor in breast carcinogenesis, accounting for an average of 5 to 10% of cases [29,38]. It is believed that family history may influence other factors, including tumor stage and grade [38]. However, in our study, 78.53% of patients did not have cases of breast cancer in their family, indicating the association of other factors in the development of cancer in these patients.

The lack of knowledge of screening methods and self-examination practices in women detected in the Al-Dhahira population is a concerning characteristic [39]. Our study demonstrated that the majority of breast cancer patients perform self-examination and mammography. This characteristic is crucial for the earlier diagnosis of this neoplasm, assisting in the appropriate therapeutic direction for each specific case [40].

The molecular classification of breast cancer elucidates important issues for the treatment of the disease, such as prognosis and therapeutic alternatives [41]. Regarding the characterization of histological and molecular types of breast cancer, we observed that the majority of patients had invasive ductal carcinoma and triple-negative breast cancer, respectively. Invasive ductal carcinoma accounts for about 50% to 80% of diagnosed breast cancer cases [29,42].

Invasive breast cancers encompass a wide spectrum of tumors that exhibit heterogeneity in clinical and morphological characteristics [33]. Triple-negative breast cancer accounts for over 15–20% of all breast cancers and, by being negative for hormone receptors, becomes therapeutically challenging due to its low response to treatment and highly invasive nature, resulting in a worse prognosis for affected patients [31,43].

Studies suggest the plausible role of oncoviruses in the pathogenesis of breast cancer [44,45]. Different studies have reported the presence of HPV in percentages ranging from 1.1% to 86% [34,46,47,48,49]. In our study, we observed a prevalence of 35.71% of the virus in breast tumors, which falls within the wide spectrum of reported prevalence.

Despite studies showing significant percentages of virus detection, other studies have failed to even identify the presence of the virus [50,51]. The variability in percentage associated with the presence of HPV in breast tissue may be related to variable sampling and the diversity of tissue processing protocols applied, in addition to the variable prevalence of HPV in different populations worldwide [20,49,52,53].

We associated molecular and histological types with the presence of HPV and observed that the triple-negative type was more frequent in invasive ductal carcinomas (77.77%). In other studies, invasive ductal carcinoma was the most prevalent subtype of breast cancer, accounting for 56.25% of samples associated with HPV in El-Sheik et al.’s study [29]. Other studies report the presence of HPV in this histological type with percentages ranging from 18 to 83.33% [54,55].

In our samples, we identified only one HPV-positive invasive lobular carcinoma, also classified as a triple-negative molecular type. In contrast, another study observed a frequency of 25% of invasive lobular carcinomas in breast cancer samples positive for HPV [55].

Our study classified 45% (9/20) of HPV-positive samples as triple-negative breast cancer, followed by 20% of the HER2 overexpression type, and 5% of the luminal type. It has been observed that there is a higher presence of HPV DNA in triple-negative and HER2+ breast cancers compared to the luminal types [47]. Although HPV cannot yet be associated with a specific molecular type, the data supports the idea that HPV may be related to a more aggressive cancer phenotype.

The integration of HPV into the human genome is one of the main mechanisms for the development of cancer from this virus; however, the episomal form is still found in many cases of HPV-associated cancers, indicating that both forms may be related to the carcinogenic activity of the virus [56,57]. HPV integration occurs after a break in the E2 gene, which is the main repressor of the expression of the E6 and E7 oncogenes [56]. When present at high levels, E2 binds to the p97 promoter in the long control region (LCR) of HPV, preventing RNA polymerase II from binding to p97 and consequently repressing the expression of E6 and E7 [58,59,60]. The E2/E6 ratio thus becomes a commonly used parameter to characterize the physical status of the virus [61]

One study observed that, for the majority of evaluated samples, the E2/E6 ratio was less than 1, indicating that the HPV was in the mixed form [34]. Our study quantified the E2 protein to determine the physical status of the viral genome, and in 65% of the samples, a predominance of the mixed form (episomal and integrated) of the HPV was found. Other studies have reported that the HPV genome in breast cancer primarily presented in the integrated form, ranging from 86% to 100%, while one study detected the mixed form of the HPV in 8.3% of breast tumors [12,62].

All the HPV-positive samples were type 16, one of the main oncogenic risk types of the virus [16]. The highest percentage of virus positivity associated with HPV16 has also been reported in other studies [29,55]. HPV16 has been found mainly in more advanced breast tumors, grades II and III, potentially associated with a more aggressive cancer behavior [63].

In our study, the viral load of HPV16 reached 80 copies/cell in triple-negative tumor types. These data are significantly higher than those reported by other research, which suggests that the viral load found in breast tissue is low [18,64,65]. The HPV viral load is linked to the severity of lesions and cancer progression, and despite this, studies have shown that even one copy of the HPV genome per cell is sufficient to induce neoplastic transformations caused by the virus’s oncoproteins [66].

HPV oncoproteins can interact with pathways related to cell proliferation, angiogenesis, inflammation, and metastasis [67]. The E5 oncoprotein induces evasion of the immune system by negatively regulating MHC-I, and can also stimulate EGFR signaling pathways involved in cell growth and proliferation [17,68,69]. Here, we detected high expression of E5 in HER2 and triple-negative samples, confirming not only the presence but also the activity of the virus in patients with breast cancer.

Overall, studies related to HPV-mediated carcinogenesis typically involve analyses related to the E6 and E7 proteins [48,70,71]. However, in breast cancer, some studies have failed to detect the activity of these oncoproteins [72], while others have only found low expression in cases positive for HPV [70]. In this regard, it is suggested that the oncogenic activity of the HPV in breast cancer may be related to the E5 oncoprotein.

The presence of the HPV has been associated with increased inflammatory cytokines (IL-1, IL-6, IL-17, TGF-β, TNF-α, and NF-kB) and tumor progression [34]. Studies have demonstrated that a high expression of E5 can be found in cancer at higher pre-neoplastic and neoplastic grades [73,74,75]. A study conducted with triple-negative breast cancer cells MDA-MB-231 showed that TNF-α was stimulated by E5, reinforcing the activity of this oncoprotein in breast tumor cells [25].

Our study demonstrated a significant presence of HPV DNA in breast tumor samples, confirmed by both the amplification of the virus’s L1 region and activity indicated using gene expression analyses, particularly the E5 oncoprotein. These findings underscore a potential link between HPV and aggressive breast cancer subtypes, such as invasive ductal and triple-negative tumors, suggesting HPV’s involvement in breast carcinogenesis. However, the exact role of HPV in breast cancer remains to be fully elucidated. We encourage further studies with larger sample sizes and in other regions to contribute to this elucidation.

## 5. Conclusions

This study provides a comprehensive characterization of breast cancer risk factors in patients from northeastern Brazil, who are predominantly over 56 years of age and maintain healthy lifestyles. The significant detection of HPV in these samples and the association with specific tumor types highlight the virus’s potential impact on the pathogenesis of breast cancer. The high expression of the E5 oncoprotein in HPV-positive samples further emphasizes its potential as a target for further research. To determine the mechanisms by which HPV may influence breast cancer development and to explore novel preventative, diagnostic, and therapeutic strategies, additional studies involving larger and more diverse populations are crucial.

## Figures and Tables

**Table 1:** Primers used for the detection of HPV in breast tumors.

Gene		Sequence	Size (pb)	Reference
β-globin	PC04	ACACAACTGTGTTCACTAGC	110 bp	Baldez et al. [21]
	GH20	CAACTTCATCCACGTTCACC0		
L1-HPV	MY09	CGTCCMARRGGAWACTGATC	450 bp	Manos et al. [22]
	MY11	GCMCAGGGWCATAAYAATGG		
	GP5	TTTGTTACTGTGGTAGATAC	150 bp	de Roda Husman et al. [23]
	GP6	GAAAAATAAACTGTAAATCA		

**Table 2:** Patient characteristics.

Characteristics	N(%)
**Age**	
<55	27 (48.2)
>56	29 (51.8)
**IBM**	
Underweight	2 (4.2)
Normal Weight	22 (45.8)
Overweight	14 (29.2)
Obesity	10 (20.8)
**Smoking**	
Yes	13 (23.2)
No	37 (66.1)
Previous smoker	6 (10.6)
**Co-morbidity**	
**Hypertension**	
Yes	22 (39.3)
No	34 (60.7)
**Diabetes mellitus**	
Yes	5 (8.9)
No	51 (91.1)

**Table 3:** Characteristics of patients regarding gynecological status.

Characteristics	N(%)
**Age of first menstruation**	
08–10 years old	3 (5.3)
11–14 years old	38 (67.8)
15–17 years old	14 (25.0)
Older than 18 years old	1 (1.8)
**Menopause Age**	
<40 years old	3 (5.3)
40–45 years old	6 (10.7)
45–50 years old	20 (35.7)
>50 years old	11 (19.6)
Not in menopause	16 (28.6)
**Number of pregnancy**	
1–3 children	28 (50.0)
4–7 children	14 (25.0)
>8 children	7 (12.5)
No children	7 (12.5)
**Age of first pregnancy**	
12–17 years old	6 (12.2)
18–27 years old	36 (73.5)
>28 years old	7 (14.3)
**Breastfeeding**	
Yes	31 (55.4)
No	25 (44.7)
**Use of contraceptive**	
Yes	26 (46.4)
No	30 (53.6)

**Table 4:** Characteristics of patients regarding breast cancer screening.

Characteristics	N(%)
**Self-examination**	
Yes	44 (78.6)
No	12 (21.4)
**Mammography**	
Yes	31 (55.4)
No	25 (44.7)
**Family history**	
Yes	12 (21.4)
No	44 (78.6)

**Table 5:** Characteristics of tumors in patients with breast cancer.

Characteristics	N(%)
**Histological Type**	
Ductal carcinoma in situ	8 (14.3)
Invasive Ductal carcinoma	44 (78.6)
Invasive Lobular Carcinoma	1 (1.8)
Medullary carcinoma	1 (1.8)
Mucinous carcinoma	1 (1.8)
Fibroadenoma	1 (1.8)
**Molecular type**	
Luminal	10 (17.9)
HER2-enriched	6 (10.7)
triple-negative	27 (48.2)
not-identified	13 (23.2)
**HPV infection**	
Yes	20 (35.7)
No	36 (64.3)
**Physical Status**	
Episomal	3 (15.0)
Integrated	4 (20.0)
Mixed	13 (65.0)

**Table 6:** Histological stratification of HPV-positive samples and their physical status.

Positive HPV x Histological Type									
	Ductal Carcinoma In Situ			Invasive Ductal Carcinoma			Invasive Lobular Carcinoma		
**N(%)**	4 (20.00)			15 (75.00)			1 (5.00)		
**Physical Status**	Episome	Integrated	Mixed	Episome	Integrated	Mixed	Episome	Integrated	Mixed
**N** **(%)**	0	2 (50.0)	2 (50.0)	3 (20.0)	2 (13.3)	10 9 (66.6)	0	0	1 (100.0)

**Table 7:** Distribution among the different molecular types and histological types of HPV-positive breast cancer.

	Ductal Carcinoma In Situ N (%)	Invasive Ductal Carcinoma N (%)	Invasive Lobular Carcinoma N (%)
**Luminal**	0 (0.0)	1 (11.1)	0 (0.0)
**HER2-enriched**	1 (50.0)	1 (11.1)	0 (0.0)
**Triple-negative**	1 (50.0)	7 (77.7)	1 (100.0)
**Total**	**2 (100.0)**	**9 (100.0)**	**1 (100.0)**

## Data Availability

The data are available and can be accessed by contacting the corresponding author.
